# Indolent systemic mastocytosis limited to the bone: a case report and review of the literature

**DOI:** 10.1590/1516-3180.2013.1313460

**Published:** 2013-06-01

**Authors:** Pedro Pinto-Lopes, Francisco Adão Fonseca, Roberto Silva, Pedro von Hafe, Elsa Fonseca

**Affiliations:** I MD, MSc. Doctoral Student of Molecular Medicine and Oncology, School of Medicine of the University of Porto; and Internal Medicine Resident, Hospital São João, Porto, Portugal.; II MD. Monitor of Clinical Semiotics, School of Medicine of the University of Porto; and Internal Medicine Resident, Hospital São João, Porto, Portugal.; III MD, MSc. Monitor of Biopathology, School of Medicine of the University of Porto; and Pathology Resident, Hospital São João, Porto, Portugal.; IV MD, PhD. Assistant Professor, Department of Internal Medicine, School of Medicine of the University of Porto; and Internal Medicine Specialist, Hospital São João, Porto, Portugal.; V MD, PhD. Associate Professor, Department of Pathology, School of Medicine of the University of Porto; Pathology Specialist, Hospital São João; and Consultant Researcher, Institute of Molecular Pathology and Immunology of the University of Porto (IPATIMUP), Porto, Portugal.

**Keywords:** Mastocytosis, Myeloproliferative disorders, Musculoskeletal diseases, Osteoporosis, Low back pain, Mastocitose, Transtornos mieloproliferativos, Doenças musculosqueléticas, Osteoporose, Dor lombar

## Abstract

**CONTEXT:**

Systemic mastocytosis is defined as a clonal disorder of mast cells and their precursor cells and is currently classified as a myeloproliferative neoplasm. Its clinical course has a wide spectrum, ranging from indolent disease, with normal life expectancy, to highly aggressive disease, associated with multisystemic involvement and poor overall survival. The aim of this study was to report a case of indolent systemic mastocytosis, focusing on the diagnostic challenges, with a review of the literature.

**CASE REPORT:**

A 79-year-old Caucasian woman with osteoporosis was evaluated at the Emergency Department because of complaints of low back pain. Before this, she had consulted an orthopedist and had undergone some imaging examinations, namely a bone scan that revealed a “superscan” pattern. Due to her pain complaints and these test results, the patient was admitted to the Department of Internal Medicine. After undergoing several analytical tests and some additional imaging examinations to rule out some important differential diagnoses, she then underwent bone marrow biopsy, which made it possible to identify indolent systemic mastocytosis.

**CONCLUSION:**

Systemic mastocytosis is a rare entity that is difficult to diagnose. Its symptoms are often unspecific and frequently ignored. Skeletal changes may be the first and only manifestation of the disease and in some cases, like this one, the diagnosis is made only after histological examination. The key point for the diagnosis is to contemplate the possibility of systemic mastocytosis.

## RESUMO

**CONTEXTO:**

A mastocitose sistêmica é definida como um distúrbio clonal do mastócito e suas células precursoras, sendo atualmente classificada como uma neoplasia mieloproliferativa. Seu curso clínico tem um espectro alargado, variando desde a doença indolente caraterizada por uma sobrevida normal, até a doença altamente agressiva associada a um envolvimento multissistêmico e a uma sobrevida reduzida. Este artigo reporta um caso de mastocitose sistêmica indolente, focando as principais dificuldades diagnósticas e fazendo uma revisão sistematizada da literatura.

**RELATO DO CASO:**

Mulher de 79 anos, caucasiana, com antecedentes de osteoporose, foi avaliada no Serviço de Urgência por queixas de dor lombar. Antes disso, a doente havia consultado um ortopedista e realizado alguns exames de imagem, nomeadamente uma cintilografia óssea, que revelou um padrão em “*superscan*”. Em virtude de suas queixas álgicas e dos resultados dos estudos efetuados, foi internada no Departamento de Medicina Interna. Depois de realizados vários estudos analíticos e exames de imagem suplementares para excluir algumas importantes hipóteses de diagnóstico, a doente fez uma biópsia de medula óssea que permitiu identificar uma mastocitose sistêmica indolente.

**CONCLUSÃO:**

A mastocitose sistêmica é uma entidade rara e difícil de diagnosticar. Os seus sintomas são muitas vezes inespecíficos e frequentemente ignorados. As alterações esqueléticas podem ser as primeiras e as únicas manifestações e, em certos casos, como este, o diagnóstico só pode ser feito após exame histológico. O mais importante para o diagnóstico é contemplar a hipótese de estarmos perante uma mastocitose sistêmica.

## INTRODUCTION

Mastocytosis is a stem cell-derived clonal myeloproliferative disease with manifest mast cell (MC) accumulation in one or multiple tissues.^1^^,^^2^^,^^3^ Since 2008, the World Health Organization (WHO) has included this disease in the category of myeloproliferative neoplasms (MPNs).^1^

Mastocytosis is subdivided into two groups of disorders: cutaneous mastocytosis (CM) and systemic mastocytosis (SM). The former describes forms of mastocytosis that are limited to the skin, and the latter defines forms in which mast cells infiltrate extracutaneous organs, with or without skin involvement.^2^^,^^3^ In all its forms, it is a rare disorder with unknown incidence that can occur at any age, with slight preponderance among males.^4^^,^^5^

Skeletal lesions often occur in patients with systemic mastocytosis, affecting primarily the axial skeleton, pelvis and proximal ends of long bones. Although bone pain is a relatively frequent symptom, pathological fractures are rare. Osteolysis and osteosclerosis are the most common pattern of bone involvement. These lesions can be mistaken for metastatic disease, Paget’s disease and hyperparathyroidism, among others.^6^

Patients with systemic mastocytosis may go undiagnosed for many years until the disease manifests itself in the form of skeletal lesions, due to the nonspecific nature of its symptoms.^6^ The following case report is an example of systemic mastocytosis presenting as a skeletal disorder that was diagnosed in Hospital São João, Porto, Portugal. The report focuses on the diagnostic difficulties and presents a literature review.

## CASE REPORT

A 79-year-old Caucasian female living in Covilhã (an area in the countryside) was admitted to the Emergency Department (ED) of Hospital São João in July 2011, complaining of severe low back pain.

Her significant past medical history included osteoporosis, which had been diagnosed more than 10 years earlier, without any prior reports of spontaneous fractures. She had therefore been medicated with alendronate plus cholecalciferol. She also said that she did not have any known allergies, had not been in contact with animals of the countryside and had not been drinking unpasteurized milk. The patient did not have any particular history of trauma and described the onset of the pain as gradual, with four years of evolution. During these years, she was admitted to the ED several times due to pain complaints and was then discharged, medicated with analgesic drugs, thereafter remaining asymptomatic for long periods of time. She said that she did not have any constitutional symptoms like significant weight loss, fever, fatigue, malaise, night sweating or decreased appetite.

**Figure 1.**
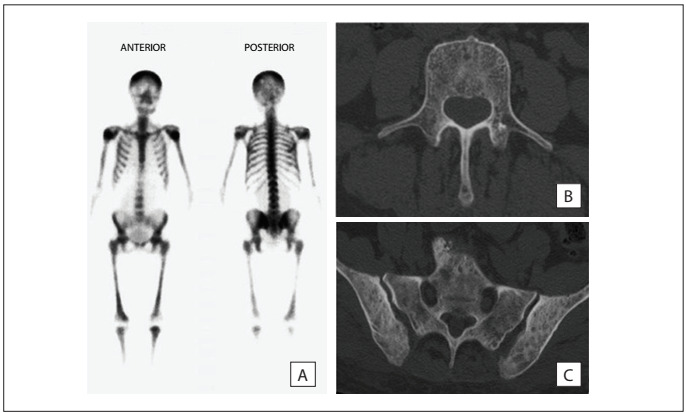
A) ^99m^Tc bone scintigraphy, showing diffusely increased skeletal uptake of the radiotracer, with minimal activity in the soft tissues and kidneys (“superscan” pattern); B) and C) Computed tomography scan at the lumbosacral level, revealing multiple discrete osteolytic and osteoblastic lesions and diffuse osteopenia, relating to the vertebrae, sacrum and iliac bone.

In May 2011, due to worsening of the low back pain, which was refractory to analgesia, she consulted an orthopedist who requested a spinal radiograph and a bone scan. The spinal radiograph showed some degree of osteopenia without any evidence of vertebral fractures. On the other hand, the bone scintigraphy revealed a “superscan” pattern **(**Figure 1A**)**, which is defined as an increase in the ratio of skeletal to renal uptake in an isotopic bone scan, with decreased or absent activity of the kidneys, despite all other evidence indicating normal renal function.

Two months later, in July 2011, as a result of severe pain complaints, the patient was once more admitted to the ED. Her physical examination was unremarkable. There were no skin abnormalities, lymphadenopathy or hepatosplenomegaly. It was decided to admit the patient to the Internal Medicine ward in order to investigate the etiology of these pain complaints, among which the main possibilities were: bone metastatic disease of unknown primary origin, multiple myeloma, Paget’s disease, hyperparathyroidism, Pott’s disease, chronic Brucella infection, sarcoidosis or systemic mastocytosis.

The initial workup included a broad laboratory assessment, which revealed mild microcytic/normochromic anemia (hemoglobin: 10.5 g/dl; normal range: 12-16); elevated lactate dehydrogenase (LDH: 419 U/l; normal range: 135-225); and low serum 25-hydroxyvitamin D (25(OH)D: 11 µg/ml; deficiency < 20 and recommended value > 30). All the other blood tests requested were within the reference range, namely: white blood cell and platelet counts, liver function tests, renal function, electrolytes, C-reactive protein, sedimentation rate and thyroid parathyroid function.

Because of the observed normal parathyroid function and phosphorus-calcium metabolism, the hypothesis of hyperparathyroidism was therefore discarded. The workup for occult malignancy and multiple myeloma was negative. It was decided to rule out several granulomatous diseases like Pott’s disease (tuberculous spondylitis), chronic brucellosis or sarcoidosis. However, all the tests performed were negative too.

In order to evaluate and further characterize the bone lesions detected previously, it was decided to request a spinal computed tomography (CT) scan at the lumbosacral level. This confirmed the presence of multiple discrete osteolytic and osteoblastic lesions and diffuse osteopenia, which involved all the vertebrae **(**Figure 1B**)**, the sacrum and the iliac bone **(**Figure 1C**)**.

**Figure 2.**
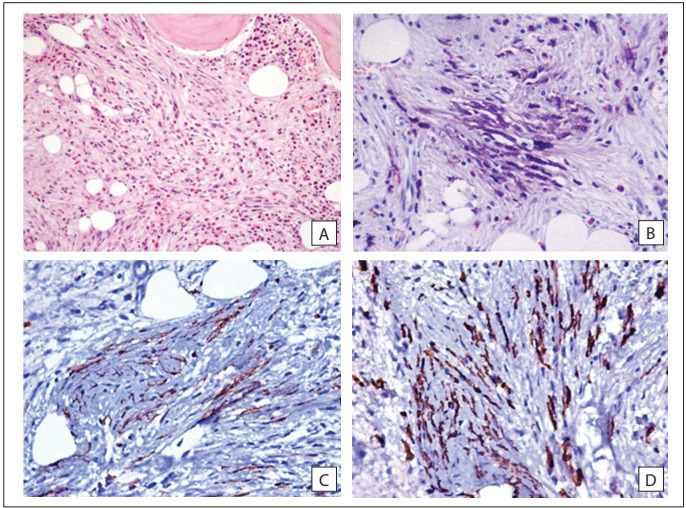
Bone marrow biopsy. A) Hypercellular marrow, with elongated mast cells, fibrosis and many eosinophils (hematoxylin and eosin staining, H&E, original magnification x 200). B) Mast cell granules are easily revealed by means of Giemsa staining (original magnification x 400). Positive c-kit C) and tryptase D) immunostaining, which confirms the mast cell nature of cells infiltrating the marrow (original magnification x 400).

The next step was to perform a bone biopsy. The histological examination showed fibrosis, hematopoietic cells, eosinophils and a significant number of mast cells, some of them exhibiting spindle-shaped morphology. No cytokeratin 8/18-positive cells were identified, thereby virtually ruling out the possibility of metastatic carcinoma. No epithelioid granuloma was identified, and the Ziehl-Neelsen staining of this tissue was negative for acid-fast bacilli. The bone biopsy, in conjunction with some other tests performed previously, practically excluded the differential diagnoses of metastatic disease, Paget’s disease, Pott’s disease, chronic brucellosis and sarcoidosis. These results motivated a subsequent evaluation, on a bone marrow biopsy. Histological examination on this material revealed hypercellular bone marrow, with areas of mast cell aggregates (confirmed by the Giemsa histochemical stain, CD117/c-kit and tryptase immunohistochemical stain). The mast cells were spindle-shaped, in a background of fibrosis and a substantial number of eosinophils **(**Figure 2**)**. This biopsy was diagnostic for systemic mastocytosis. Nonetheless, the serum tryptase level was normal (tryptase: 9.10 ng/ml; normal range: < 20 ng/ml).

The patient was discharged one month after admission, with a prescription for iron supplements and analgesics. She was also referred to a hematologist for regular evaluation and follow-up.

## DISCUSSION

Mast cells (MCs) are tissue-dwelling cells characterized by prominent cytoplasmic granules containing chemical pro-inflammatory and vasoactive mediators. They play a crucial role in allergic reactions and take part in other pathophysiological conditions such as innate and acquired immunity, wound healing, fibrosis and autoimmune diseases.^7^ MCs are derived from CD34+, CD117+ (c-kit) and CD13+ progenitors that circulate in the peripheral blood and lymphatic system and migrate into vascularized tissues. Final maturation takes place in these tissues under the influence of local factors, like the stem cell factor (SCF) and other mediators such as the interleukins IL-3, IL-4, IL-9 and IL-10, the nerve growth factor (NGF) and some chemokines. MC proliferation, differentiation and survival are stringently regulated by these factors, especially the stem cell factor (SCF) and the ligand for the c-kit tyrosine kinase growth factor receptor, which is expressed on the MC surface.^3^^,^^7^ Abnormalities in SCF regulation or the c-kit receptor permanently affect the growth, differentiation, apoptosis and activation of mast cells.^3^ Activation of mutations of the c-kit receptor leads to pathological accumulation of mast cells in tissues as a result of clonal expansion and apoptotic defects. Several c-kit mutations have been reported in cases of systemic mastocytosis, although the most common one consists of substitution of valine for aspartate in codon 816 (Asp816Val), thus resulting in constitutive activation of the c-kit receptor.^8^ These mutations are rarely found in germline cells; in fact, they are somatic in most cases.^9^^,^^10^ Familial occurrence has been reported, albeit rarely, and it remains unclear whether all of these patients had c-kit mutations.^4^

Systemic mastocytosis encompasses a group of heterogeneous myeloproliferative neoplasms (MPNs) characterized by excessive proliferation of neoplastic MCs that accumulate in one or more organs, particularly in hematopoietic tissues. According to the 2008 WHO Classification of Tumors of Hematopoietic and Lymphoid Tissues, it can be categorized into several subtypes such as: indolent systemic mastocytosis (ISM), systemic mastocytosis with an associated clonal hematologic non-mast cell lineage disease (SM-AHNMD), aggressive systemic mastocytosis (ASM), mast cell leukemia and mast cell sarcoma.^4^^,^^11^

Patients with systemic mastocytosis may have symptoms relating to involvement of the hematopoietic system, gastrointestinal system, skin and immune system, as well as in association with coexisting hematological diseases. Laboratory examination may reveal a serum tryptase level of 20 ng/ml or higher, which would be suggestive of systemic mastocytosis, although normal levels do not rule this out.^4^^,^^12^

One additional evaluation that may be conducted because of the presentation is bone scintigraphy. In the particular case of our report, the patient presented a bone scan with a “superscan” pattern. This condition has low specificity and has been reported in cases of diffuse metabolic diseases (hyperparathyroidism, osteomalacia, Paget’s disease, renal osteodystrophy and hypervitaminosis D), hematological diseases (multiple myeloma, lymphoma, systemic mastocytosis, aplastic anemia, myelofibrosis, leukemia and Waldenström macroglobulinemia), fibrous dysplasia and metastatic carcinoma, particularly from the prostate or breasts, and less commonly from the bladder or lungs.^13^

Although the diagnosis of systemic mastocytosis is generally suspected on the basis of the clinical history and physical findings, and supported by laboratory procedures, it can only be established through a histological diagnosis.^4^ Bone marrow examination also assists in diagnosing systemic mastocytosis associated with a clonal hematologic non-mast cell lineage disease (SM-AHNMD). This is important, because the long-term prognosis of these patients relies heavily on the associated hematological malignancy.^14^

**Chart 1.**
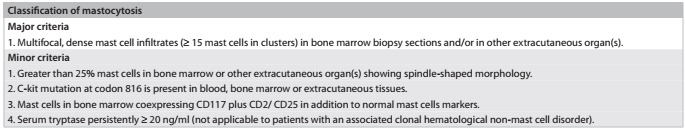
World Health Organization diagnostic criteria for systemic mastocytosis*

The diagnosis of SM is based on a set of diagnostic criteria **(**Chart 1**)**, requiring the presence of one major criterion plus one minor criterion or a combination of three minor diagnostic criteria.^3^^,^^4^^,^^14^ In this case report, one major criterion [multifocal dense infiltrates of MCs in bone marrow or other extracutaneous organs (> 15 MCs aggregating)] and one minor criterion [MCs in bone marrow or other extracutaneous organs with an abnormal (spindling) morphology (> 25%)] were fulfilled.

*A diagnosis of systemic mastocytosis requires fulfillment either of one major criterion plus one minor criterion or of three minor criteria.

**Chart 2.**
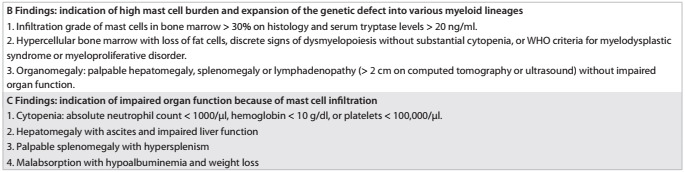
B and C findings in systemic mastocytosis, used to assess disease burden and disease aggressiveness

Once a diagnosis of SM has been made, patients are categorized further according to the presence of “B” and “C” findings, which assess disease burden and disease aggressiveness, respectively **(**Chart 2**)**.^11^ Patients who have systemic mastocytosis with no “B” or “C” findings are categorized as cases of ISM, as in this particular case. Those who have “B” findings are categorized as presenting smoldering systemic mastocytosis (SSM), a subtype of ISM that has a more aggressive clinical course; and those who have “C” findings are categorized best as presenting ASM.^11^

We conducted a systematic search in the main electronic databases (PubMed, Embase, Scopus, Scirus, Lilacs, Cochrane Library and SciELO), to find papers that reported the difficulties and challenges of the clinical approach towards ISM. In order to make the search as wide as possible, no limits were applied regarding the date of publication, the language used or the type of article **(**Table 1**)**.

**Table 1.** Systematic review of the literature performed on July 10, 2012

Electronic databases	Search strategy	Results	
		Found	Related
PubMed	“Mastocytosis” [MeSH] AND “Musculoskeletal Diseases” [MeSH] *Limits: Humans*	216 papers	12 original articles 15 reviews 24 case reports 1 letter
Scopus	“Mastocytosis” AND “Bone Diseases”	85 papers	6 original articles 17 reviews 9 case reports 2 letters
Embase	“Mastocytosis” AND “Bone Diseases”	9 papers	1 letter 1 review 1 case report
Cochrane Library	“Mastocytosis”	23 papers	-
Lilacs	“Mastocytosis” [DeCS]	83 papers	6 reviews 2 case reports
SciELO	“Mastocytosis” [DeCS]	20 papers	4 reviews
Scirus	“Mastocytosis” AND “Bone Diseases”	6 papers	1 case report

MeSH = Medical Subject Headings; DeCS = Descritores em Ciências da Saúde.

With regard to treatment, patients presenting systemic mastocytosis with mediator-related symptoms should be prescribed mediator-targeting drugs (such as antihistamines, cromolyn sodium, anti-leukotriene agents, proton pump inhibitors or bisphosphonates), and these should avoid triggering stimuli that might cause mediator release from MCs.^3^^,^^4^^,^^15^

Omalizumab (anti-IgE), a humanized murine monoclonal antibody that inhibits binding of IgE to mast cells, can theoretically reduce the anaphylaxis in patients with systemic mastocytosis, and some recent studies have shown beneficial effects.^16^^,^^17^ This application of omalizumab has not been approved by the Federal Drug Administration (FDA) and requires further study.^15^^,^^16^^,^^17^

Patients with ISM rarely require cytoreductive therapy and have an excellent long-term prognosis, with only 1-5% experiencing progression to more aggressive forms of the disease. Therefore, yearly monitoring is appropriate for uncomplicated patients, although the frequency of evaluation should be increased if the symptoms worsen or new symptoms appear. For patients with SM-AHNMD, separate treatment plans for the SM and AHNMD components of the disease should be established.^3^ Those with ASM and mast cell leukemia (MCL) may have some benefit from cytoreductive therapies (e.g. interferon alpha-2b or cladribine), although the disease usually recurs and the long-term prognosis is poor.^15^ Use of targeted drugs like tyrosine kinase inhibitors may be appropriate for some patients with SM-AHNMD, ASM or MCL, and these inhibitors sometimes achieve effective suppression of constitutively activated c-kit and may result in killing of KIT-mutated mast cells.^18^ Imatinib, for instance, is an agent that preferentially kills mast cells with wild-type KIT, and therefore most patients with systemic mastocytosis are not candidates for imatinib therapy because most of them have the D816V KIT mutation, which confers resistance to imatinib. Hence, this drug is not expected to be used widely to treat SM.^3^^,^^19^ Masitinib, like imatinib, is also a tyrosine kinase inhibitor with activity against wild-type but not against D816V-mutant kit.^20^ Clinical trials on dasatinib, a drug that inhibits the kinase activity of KIT D816V with comparable efficacy in inhibiting the activity of wild-type KIT, have generally been disappointing.^21^ There are currently no curative therapies for SM, and the aim of the treatment is to reduce symptoms and improve quality of life.

## CONCLUSION

The present report describes a case of systemic mastocytosis that was diagnosed only after a histological examination. Mastocytosis is a rare condition with symptoms that may be ignored and considered insignificant by physicians, especially in indolent cases like this. As demonstrated in our patient, the skeletal changes seen in mastocytosis may be the first and only recognized manifestation of the disease, thus bringing the patient to the attention of the family doctor or an orthopedist who usually has limited exposure to such cases. Quite often, mastocytosis may be mistaken for other systemic or metastatic diseases. Therefore, better awareness of this disease and its characteristic clinical features is an essential step towards making an accurate diagnosis. The key point in diagnosing systemic mastocytosis is to contemplate the possibility of systemic mastocytosis.
